# Chromosomal diversification in ribosomal DNA sites in *Ancistrus* Kner, 1854 (Loricariidae, Ancistrini) from three hydrographic basins of Mato Grosso, Brazil

**DOI:** 10.3897/CompCytogen.v5i4.1757

**Published:** 2011-11-09

**Authors:** Sandra Mariotto, Liano Centofante, Marcelo Ricardo Vicari, Roberto Ferreira Artoni, Orlando Moreira-Filho

**Affiliations:** 1 Instituto Federal de Educação, Ciência e Tecnologia de Mato Grosso, campus Bela Vista, Cuiabá, Mato Grosso, Brazil; 2 Instituto de Biociências, Universidade Federal do Mato Grosso, Cuiabá, Mato Grosso, Brazil; 3 Departamento de Biologia Estrutural, Molecular e Genética, Universidade Estadual de Ponta Grossa, Ponta Grossa, Paraná, Brazil; 4 Programa de Pós-Graduação em Genética e Evolução, Universidade Federal de São Carlos, São Carlos, São Paulo, Brazil

**Keywords:** karyotype evolution, Robertsonian rearrangement, Ag-NORs, 18S rDNA, 5S rDNA

## Abstract

Populations of seven *Ancistrus* species were analyzed from streams and rivers of three hydrographic Brazilian basins. All populations showed different diploid numbers (2n), fundamental numbers (FNs), and karyotypes. Some representatives of Loricariidae have 2n = 54 chromosomes, which is very likely an ancestral cytotaxonomic characteristic, but many other representatives show extensive karyotype diversification. In the *Ancistrus* species studied, extensive karyotypic differentiation, which is generally associated with chromosome number reduction and rearrangement of the ribosomal RNA gene (rDNA) sites, was verified. Chromosomal locations of 18S and 5S rDNA were jointly detected using fluorescence *in situ* hybridization (FISH). In all the *Ancistrus* species analyzed, 18S rDNA sites were detected only on one chromosome pair, though this differed among species. 5S rDNA was located on 1–3 chromosome pairs either separately or in synteny with 18S rDNA in four of the seven species/populations. Hence the karyotype differentiation in *Ancistrus* species could be associated with a morphological speciation process, suggesting that chromosome fusions, inversions, deletions, duplications, and heterochromatination could contribute to the karyotype evolution of these neotropical armored catfishes.

## Introduction

In eukaryotes, 5S and 18S ribosomal genes (rDNA) are arranged into two distinct classes, namely the major rDNA family composed of 18S, 5.8S, and 28S genes and the minor family composed of 5S genes (Long and David 1980, Pendás et al. 1994). Silver nitrate-stained nucleolus organizing regions (Ag-NORs) have long been used in cytotaxonomic analysis of fish (Galetti 1998). However, cytogenetic comparisons of banding patterns prove inadequate when dealing with species with highly rearranged genomes or with other highly divergent species (Chowdhary and Raudsepp 2001). Hence, comparison using fluorescence *in situ* hybridization (FISH) associated with classical chromosomal markers has become the preferred method for genome comparisons at the cytogenetic level because it allows complete chromosome probes of a species to be hybridized *in situ* with chromosomes of other species, thereby allowing the detection of homologous genomic regions (Vicari et al. 2010; Bellafronte et al. 2011; Machado et al. 2011).

Studies that characterize the chromosomal locations of 5S and 18S rDNA in Siluriformes are scarce (Kavalco et al. 2004; Centofante et al. 2006; Mendes-Neto et al. 2011). A few such studies were conducted in Pimelodidade and Pseudopimelodidae, where non-syntenic 5S and 18S ribosomal regions were observed (Carvalho and Dias 2007; Garcia and Moreira-Filho 2008; Marques et al. 2008; Matoso et al. 2011; Moraes Neto et al. 2011; Silva et al. 2011). In Loricariidae, chromosomes with syntenic 5S and 18S regions were observed in some groups like Neoplecostominae and the out group Trichomycteridae (Ziemniczak 2011).

The classification of subfamilies within Loricariidae and the genera relationships have been targets of repeated reformulation (Isbrücker 1980; Armbruster 2004; Reis et al. 2006). Armbruster (2004) placed the subfamilies Hypoptopomatinae, Hypostominae, Lithogeneinae, Loricariinae, and Neoplecostominae as valid groups within this family. In this revision, the former subfamily Ancistrinae is regarded as a synonym of Hypostominae, which comprises five tribes, namely Corymbophanini, Rhinelepini, Hypostomini, Pterygoplichthini, and Ancistrini. The available karyotypic data on Loricariidae show a high diversity of diploid numbers (2n) and chromosomal features, although some evolutionary trends can be defined among the distinct subfamilies (Ziemniczak 2011). Artoni and Bertollo (2001) stated that 2n = 54 chromosomes would be a putative plesiomorphic trait in Loricariidae once it is reported in the basal genera and the sister group of the superfamily Loricarioidea, as described in Trichomycteridae. Therefore, groups such as Loricariinae and Hypostominae present a wide diversity of 2n and chromosomal markers, comprising highly differentiated traits in relation to primitive features of Loricariidae (Artoni and Bertollo 2001; Mariotto et al. 2009). These inferences based on cytogenetic data are in agreement with the last hypotheses of morphological (Armbruster 2004; Reis et al. 2006) and molecular phylogenies (Cramer et al. 2011) in this family.

Among Hypostominae, few species maintain the number of 54 chromosomes. All representatives in Ancistrini have 2n ≤ 54, indicating that centric fusions contributed to the karyoevolution of this tribe. In this study, we used comparative chromosomal markers to establish chromosome homologies among some *Ancistrus* species and investigated the cytotaxonomical, biogeographical, and karyoevolutionary features of this group.

## Materials and methods

One hundred and thirty six specimens [male (M) and female (F)] of seven *Ancistrus* species were cytogenetically analyzed. All specimens were from rivers and streams of three hydrographic basins (a, b, and c) of Mato Grosso state, Brazil: (a) Paraguay basin, Coxipó river, 15°21'59"S, 55°57'11"W, *Ancistrus claro* Knaack, 1999 (11 M and 10 F);Sepotuba river, 14°41'35"S, 57°48'14"W, *Ancistrus* sp. 04 (12 M and 15 F); Currupira river, 15°07'59"S, 56°49'47"W, *Ancistrus* sp. 08 (7 M and 8 F); Flechas stream, 15°58'7"S, 57°19'7"W, Fundo stream, 16°14'17"S, 56°37'31"W, and Pari stream, 15°36'6"S, 56°12'19"W, *Ancistrus* cf. *dubius* Eigenmann and Eigenmann, 1889 (2 M and 2 F from each locality); Arrombado bay, 16°21'21"S, 56°27'55"W, *Ancistrus cuiabae* Knaack, 1999(15 M and 15 F); (b) Araguaia–Tocantins basin, Salgadinho stream, 14°40'14"S, 52°21'50"W, *Ancistrus* sp. 13 (11 M and 6 F); and (**c**) Amazon basin, Matrixã river, 10°3'7"S, 57°36'27"W, *Ancistrus* sp. 06 (9 M and 5 F).

Specimens were morphologically identified and deposited in the Museu de Ciências da Pontíficia Universidade Católica do Rio Grande do Sul (MCP/PUC; MCP 41966, 41968, 41971, 41973, 41975, 41978, 41979) and Núcleo de Pesquisas Limnológicas da Universidade Estadual de Maringá, Paraná (NUPELIA/UEM; NUP 6827, 7492). *Ancistrus* sp. 04, 06, 08, and 13 present discriminative morphological characteristics that have not yet been described.

Chromosomal preparations were obtained from anterior kidney cells using an *in vivo* treatment with colchicine (Bertollo et al. 1978). The nucleolar organizing regions (NORs) were located using colloidal silver nitrate (Howell and Black 1980). A digital camera with an 8.1 Mp resolution was used in light field microscopy to photograph the Ag-NORs.

FISH was performed according to Pinkel et al. (1986). Two probes were used, namely an 18S rDNA probe obtained from the nuclear DNA of *Prochilodus argenteus* Spix and Agassiz, 1829 (Hatanaka and Galetti 2004) and a 5S rDNA probe obtained from the genomic DNA of *Leporinus elongatus* Valenciennes, 1850 (Martins and Galetti 1999). The 18S and 5S rDNA probes were labeled with biotin-16-dUTP and digoxigenin-11-dUTP, respectively, through nick translation according to the manufacturer's instructions (Roche Applied Science). The overall hybridization procedure was performed under high-stringency conditions (2.5 ng/µL from each probe, 50% deionized formamide, 10% dextran sulfate, 2×SSC, pH 7.0–7.2, incubation at 37°C overnight). After hybridization, the slides were washed in 15% formamide/0.2×SSC at 42°C for 20 min, 0.1×SSC at 60°C for 15 min, and 4×SSC/0.05% Tween at room temperature for 10 min, with the latter consisting of two washes of 5 min each. Signal detection was performed for 1 h using conjugated avidin–fluorescein isothiocyanate (Sigma) for the 18S rDNA and anti-digoxigenin–rhodamine (Roche Applied Science) for 5S rDNA at 1:1000 and 1:200 dilutions, respectively, in non-fat dry milk buffer (5% non-fat dry milk in 4×SSC). The chromosomes were counterstained with DAPI and analyzed under an epifluorescence microscope (Olympus BX41) coupled to an image capturing system (Olympus DP71). Approximately 30 metaphases were analyzed to determine the 2n, karyotypic formulae, and the presence or absence of rDNA sites on the chromosomes.

## Results

**Figure 1.**
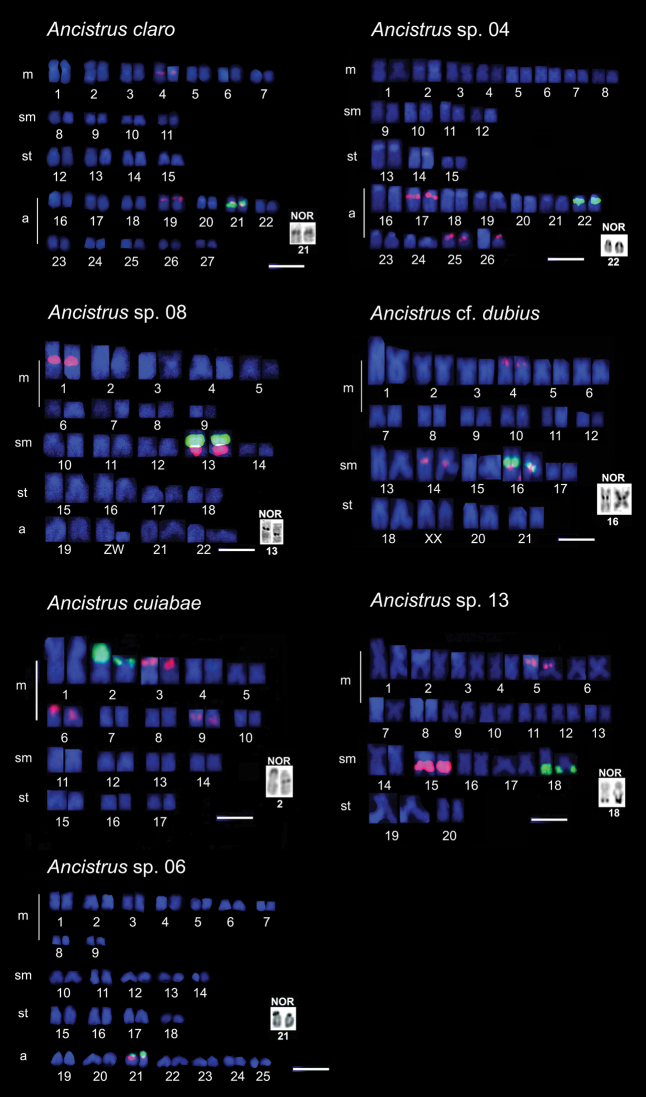
Chromosomes of *Ancistrus* species after dual color-FISH showing 5S rDNA (red) and 18S rDNA (green) sites. Silver nitrate-stained nucleolar organizing region (Ag-NOR) patterns are shown in the boxes. Bars = 10 µm.

**Figure 2.**
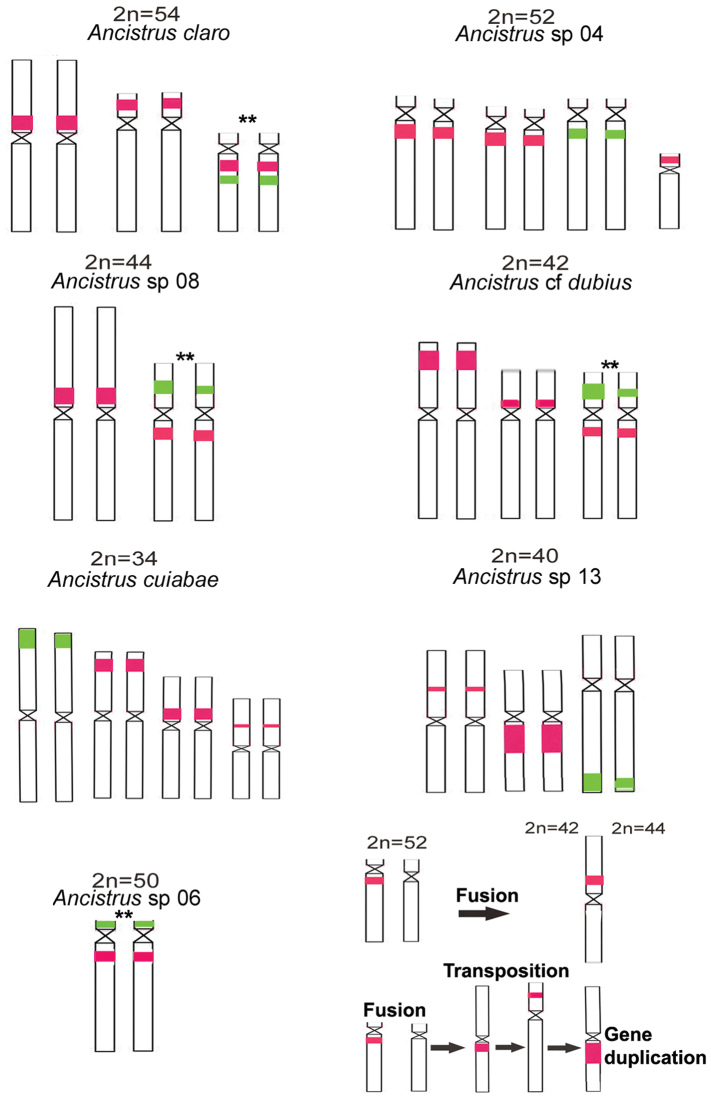
Idiograms of chromosomes bearing 5S (red) and 18S (green) rDNA (a–g); (h) probable chromosomal rearrangements (fusions, transpositions, and gene duplication) occurred during the evolution of *Ancistrus* species; ** denotes possible homeologous chromosomes.

The studied species showed variations in 2n and in karyotypic formulae (Table 1, Fig. 1). The 2n ranged from 54 chromosomes in *Ancistrus claro* to 34 chromosomes in *Ancistrus cuiabae*. The fundamental number (FN) varied from 68 to 86 chromosome arms (Table 1). NORs were seen in a single chromosome pair in all the *Ancistrus* sp. analyzed using silver nitrate staining and FISH with 18S rDNA probe (Fig. 1). However, an interspecific variation was observed in the NOR-bearing chromosome pairs and NOR locations in these chromosomes (Table 1, Fig. 1 and 2).

**Table 1.** Chromosomal data in analyzed *Ancistrus* species.

**Species/basin**	**2n**	**Karyotypic formulae**	**FN**	**SC**	**rDNA synteny**
***Paraguay basin***					
*Ancistrus claro*	54	14m+8sm+8st+24a	84	-	Present
*Ancistrus* sp. 04	52	16m+8sm+6st+22a	82	-	Absent
*Ancistrus* sp. 08	44	18m+10sm+8st+8a	80	ZZ/ZW	Present
*Ancistrus* cf. *dubius*	42	24m+10sm+8st	84	XX/XY	Present
*Ancistrus cuiabae*	34	20m+8sm+6st	68	-	Absent
***Araguaia–Tocantins basin***					
*Ancistrus* sp. 13	40	26 m+10sm+4st	80	-	Absent
***Amazon basin***					
*Ancistrus* sp. 06	50	18m+10sm+8st+14a	86	-	Present

Diploid number (2n), metacentric (m), submetacentric (sm), subtelocentric (st), acrocentric (a), fundamental number (FN), sex chromosome system (SC).

The number of 5S rDNA sites varied among species (Fig. 1). Multiple locations were observed in all species, except *Ancistrus* sp. 06 (Fig. 1). Dual-color FISH using 5S and 18S probes showed co-localized sites on apparently homeologous chromosome pairs in *Ancistrus claro*, *Ancistrus* sp. 08, *Ancistrus* cf. *dubius*, and *Ancistrus* sp. 06 (Fig. 1). However, in *Ancistrus* sp. 04, *Ancistrus cuiabae*,and *Ancistrus* sp. 13, 18S and 5S rDNA probing revealed different chromosome pairs carrying 18S and 5S rDNA (Fig. 1). *Ancistrus claro* showed syntenic rDNA site in pair 21 and four additional 5S rDNA sites (Fig. 1). In *Ancistrus* sp. 04, syntenic 18S and 5S rDNA classes in pair 22 and additional 5S rDNA sites in pairs 17 and 25, as well as one homologue in pair 26 were visualized (Fig. 1). *Ancistrus* sp. 08 showed 18S rDNA syntenic to 5S rDNA in pair 13 and an additional 5S rDNA site in pair 1 (Fig. 1). In addition, *Ancistrus* sp. 08 had a heteromorphic ZZ/ZW sex chromosome system in pair 20 (Fig. 1). *Ancistrus* cf. *dubius* showed syntenic 18S and 5S rDNA classes in pair 16, and additional 5S rDNA sites in pairs 4 and 14 (Fig. 1). This species presented an extensive heterochromatic region in pair 19 of females and in one of the complements of pair 19 of males, thus characterizing a sexual chromosomal system, XX/XY (data not shown). *Ancistrus cuiabae* showed 18S rDNA located in pair 2 and three different pairs (3, 6, and 9) carrying 5S rDNA sites (Fig. 1). In *Ancistrus* sp. 13, 18S rDNA was located in pair 18, and 5S rDNA was located in pairs 5 and 15 (Fig. 1). *Ancistrus* sp. 06 showed syntenic rDNA classes in pair 21 (Fig. 1).

## Discussion

The catfish Loricariidae is one of the most speciose components of neotropical freshwater fish fauna. The karyotypic differentiation of Ancistrini is correlated with the great diversification of forms in this tribe and may play an important role in the genetic/reproductive isolation of species (Ziemniczak 2011). Some representatives of Loricariidae have 2n = 54 chromosomes, which is very likely an ancestral cytotaxonomic characteristic, but many other representatives show extensive karyotype diversification (Artoni and Bertollo 2001; Milhomem et al. 2010; Bueno et al. 2011; Ziemniczak 2011). *Ancistrus* is the most speciose genus in the tribe and exhibits extensive karyotypic differentiation, generally associated with a chromosome number reduction (Alves et al. 2003; Mariotto et al. 2009).

This study revealed that chromosome fusion is the major mechanism in 2n reduction of some Ancistrini species. In the sister group Hypostomini, in which all species present 2n ≥ 54, it has been postulated that the increase in the subtelocentric/acrocentric chromosome number is directly proportional to 2n, thereby indicating that centric fissions have played a key role in karyotype evolution of the group (Artoni and Bertollo 2001; Milhomem et al. 2010). Although this hypothesis is partially supported in this tribe, it was not possible to correlate 2n with the proportion of subtelocentric/acrocentric chromosomes in some species (Bueno et al. 2011). In this study, we hypothesized that the 2n primitive to the family is conserved in *Ancistrus claro*, and extensive chromosomal rearrangements, such as chromosome fusions, inversions, deletions, duplications, and heterochromatination, could contribute to the chromosomal differentiation of Ancistrini. This assumption is corroborated by the NF value (Table 1), which is not maintained solely by chromosome fusion.

Ag-NORs can also be used as efficient markers in Loricariidae. A single NOR pair in an interstitial location is considered a primitive characteristic in Loricariidae and is maintained in most Ancistrini species (Artoni and Bertollo 2001; Alves et al. 2003; Mariotto et al. 2004; Mariotto and Miyazawa 2006; de Oliveira et al. 2006, 2007, Mariotto et al. 2009; Mendes Neto et al. 2011). Utilizing rDNA probes for dual-color FISH experiments have provided important information about the chromosomal diversification of this fish group. Ziemniczak (2011) described syntenic, adjacent, and interstitial locations of 18S and 5S rDNA classes in a single chromosome pair in the basal genera of Loricariidae and in its out group, Trichomycteridae. Based on these data, Ziemniczak (2011) inferred that the synteny between both rDNA classes in a single chromosome pair is a primitive condition for Loricariidae.

*Ancistrus claro*, *Ancistrus* cf. *dubius*, *Ancistrus* sp. 08, and *Ancistrus* sp. 06 conserve the interstitial NORs in a putative homologous pair. However, the chromosomal morphologies of NOR-bearing chromosomes vary, probably because of the accumulation of adjacent heterochromatin (Vicari et al. 2006, 2008; Kantek et al. 2009) and/or by variation in the size of rDNA from unequal crossover. The latter mechanism can also explain18S rDNA polymorphism in *Ancistrus cuiabae*. Based on these data, it can be said that the proposal of an ancestral karyotype in the genus is similar to that presented by *Ancistrus claro*, which has 2n = 54 chromosomes, a high NF value, and one chromosome pair with syntenic 5S and 18S rDNA classes.

FISH mapping of 5S rDNA in *Ancistrus* species showed variations in the number and shape of chromosomes bearing this ribosomal family. Most sites were observed in the interstitial portion of the long or short arm of chromosomes; however, in some cases, such as *Ancistrus* cf. *dubius* and *Ancistrus* sp. 08, pericentromeric 5S rDNA sites were visualized. The occurrence of multiple and variable 5S rDNA can be considered an important process underlying this huge karyotypic diversity. In the subfamily Loricariinae, Ziemniczak (2011) demonstrated that interstitial telomeric sites and 5S rDNA can be observed in fusion chromosomes, implying that rDNA could serve as a breakpoint for fusion in *Rinelocaria lima*. Thus, the mechanism generating fused chromosomes or others by sequence transpositions that promote chromosome diversification can help to explain karyotypic evolution in *Ancistrus* species.

Based on the trends in the karyotype evolution in Ancistrini, it can be inferred that the variation in 2n (54–34 chromosomes) in the *Ancistrus* species studied could possibly involve several chromosomal rearrangements and gene flow restriction in different hydrographic basins or rivers (Fig. 2). The 2n primitive was found in *Ancistrus claro* from the Paraguay basin, which presents syntenic 18S and 5S rDNA sites in pair 21 and no sex chromosome heteromorphism. However, this species presents two additional 5S rDNA sites, a characteristic considered apomorphic in this family. *Ancistrus* sp. 04 from the Paraguay basin presents 2n = 52 chromosomes, no syntenic rDNA sites, no sex chromosome heteromorphism, and additional 5S rDNA sites. Hence, rDNA translocation and fusion chromosomes could have occurred in species diversification, and the broken condition of the syntenic rDNAs could have originated in one lineage with *Ancistrus cuiabae* and *Ancistrus* sp. 13. The similarity among them is maintained by no syntenic rDNA sites, no sex chromosome heteromorphism, and closely hydrographic basins. However, the pair carrying 18S rDNA in *Ancistrus* sp. 04 is apparently homeologous to chromosome pair 18 in *Ancistrus* sp. 13, which could have originated in the second pair in *Ancistrus cuiabae* by chromosome fusion.

The other lineage consists of species that retain the syntenic rDNA sites (*Ancistrus claro*, *Ancistrus* sp. 08, *Ancistrus* cf. *dubius*, and *Ancistrus* sp. 06). In addition to *Ancistrus claro*, species from the Amazon basin (*Ancistrus* sp. 06) have 2n = 50 chromosomes, considered to have been derived from the family. However, this species retains a primitive single NOR pair with syntenic 5S rDNA site and no additional site. Thus, the chromosome number reduction in *Ancistrus* sp. 06 is attributable to chromosome fusion. *Ancistrus* sp. 08 and *Ancistrus dubius* from the Paraguay basin have chromosome number reduction (2n = 44 and 2n = 42, respectively) by fusion and independent pathways to differentiated sex chromosome systems (Mariotto et al. 2004; Mariotto and Miyazawa 2006). *Ancistrus* sp. 08 shows a ZZ/ZW system relative to pair 20 (Mariotto and Miyazawa 2006), whereas *Ancistrus dubius* has a XX/XY heteromorphic sex system in pair 19 (Mariotto et al. 2004).

Nevertheless, there is large chromosome plasticity among the species from the Paraguay basin, and the diversity of chromosome types with 5S and 18S ribosomal cistrons in *Ancistrus* sp. explain the high degree of karyotypic diversification in this taxon. Also, the 18S rDNA marker, which is mostly considered to be conserved in a single chromosome pair in the interstitial position, showed different site locations in different types of chromosomes.

The variation observed in the 2n, FN, and rDNA sites of the *Ancistrus* sp. could be attributed to structural and numeric chromosome rearrangements. The karyotypic data presented here are important tools for taxonomy of *Ancistrus* species. The karyotype differentiation in Ancistrini could be associated with a morphological speciation process, suggesting that chromosome fusions, inversions, deletions, duplications, and heterochromatination could contribute to the chromosomal differentiation of Ancistrini.
